# The New Year and the Physician

**Published:** 1883-01-20

**Authors:** 


### *THE NEW YEAR AND THE PHYSICIAN.*

As we go to press the New Year is already more than two weeks advanced, How rapid is the flight of time, and how swiftly moving seems the panorama of events as we look back upon our past lives! Many of the cares and responsibilities through which, as medical men, we have passed, seem not so great, perhaps, now that they are over; yet none but the physician knows how trying and arduous is the life which he leads. The emergencies of practice: its hardships, grave responsibilities, the scenes of agony and of grief and the wails of bereaved friends, who trusted to his skill and vainly appealed to him for relief and for life itself—these are the trials which, to the humane and conscientious physician, impart that subdued look, premature decline and melancholy aspect so often seen in the practitioner. This is the dark side of the picture. The true, honest and progressive practitioner has always the consciousness of having performed his duty well, and that, though he has often failed to save life where the best human skill was impotent, yet he has cured, or assisted nature in the cure of many cases, while others—not curable—have been relieved, the anguish of friends appeased and the dying pillows of those whom the Father had called up higher have been made soft and easy.

This we say of the true, honest and *progressive* physician—progressive because it is the conscientious duty of every practitioner to study his profession well, to read the journals and to keep pace with all the advances in the science, that he may be able to do the *best thing* for his patient that is known to the profession in his day.

Let, then, our brethren take courage, and if they have not heretofore acted upon these principles, let them form new and good resolves for the present year. And may brotherly love and a progressive spirit prevail throughout our ranks, and may peace, success and prosperity attend and crown the efforts of the readers of the RECORD in 1883. W.

